# Bufalin Reverses Resistance to Sorafenib by Inhibiting Akt Activation in Hepatocellular Carcinoma: The Role of Endoplasmic Reticulum Stress

**DOI:** 10.1371/journal.pone.0138485

**Published:** 2015-09-18

**Authors:** Bo Zhai, Fengli Hu, Haijiang Yan, Dali Zhao, Xin Jin, Taishi Fang, Shangha Pan, Xueying Sun, Lishan Xu

**Affiliations:** 1 Department of General Surgery, the Fourth Affiliated Hospital of Harbin Medical University, Harbin, Heilongjiang, China; 2 Department of Gastroenterology, the Fourth Affiliated Hospital of Harbin Medical University, Harbin, Heilongjiang, China; 3 Department of General Surgery, the First Affiliated Hospital of Harbin Medical University, Harbin, Heilongjiang, China; Taipei Medicine University, TAIWAN

## Abstract

Sorafenib is the standard first-line therapeutic treatment for patients with advanced hepatocellular carcinoma (HCC), but its use is hampered by the development of drug resistance. The activation of Akt by sorafenib is thought to be responsible for this resistance. Bufalin is the major active ingredient of the traditional Chinese medicine *Chan su*, which inhibits Akt activation; therefore, *Chan su* is currently used in the clinic to treat cancer. The present study aimed to investigate the ability of bufalin to reverse both inherent and acquired resistance to sorafenib. Bufalin synergized with sorafenib to inhibit tumor cell proliferation and induce apoptosis. This effect was at least partially due to the ability of bufalin to inhibit Akt activation by sorafenib. Moreover, the ability of bufalin to inactivate Akt depended on endoplasmic reticulum (ER) stress mediated by inositol-requiring enzyme 1 (IRE1). Silencing IRE1 with siRNA blocked the bufalin-induced Akt inactivation, but silencing eukaryotic initiation factor 2 (eIF2) or C/EBP-homologous protein (CHOP) did not have the same effect. Additionally, silencing Akt did not influence IRE1, CHOP or phosphorylated eIF2α expression. Two sorafenib-resistant HCC cell lines, which were established from human HCC HepG2 and Huh7 cells, were refractory to sorafenib-induced growth inhibition but were sensitive to bufalin. Thus, Bufalin reversed acquired resistance to sorafenib by downregulating phosphorylated Akt in an ER-stress-dependent manner via the IRE1 pathway. These findings warrant further studies to examine the utility of bufalin alone or in combination with sorafenib as a first- or second-line treatment after sorafenib failure for advanced HCC.

## Introduction

Hepatocellular carcinoma (HCC) is the sixth most common cancer worldwide, with more than 700,000 new cases and 700,000 deaths annually [1]. Most HCC patients are diagnosed at the advanced stages; therefore, the need for new systemic therapies is urgent. However, HCC is notoriously resistant to systemic chemotherapy [2]. Sorafenib, a multikinase inhibitor, is the first and only drug that has been approved as the standard first-line systemic treatment for advanced HCC. However, drug resistance to sorafenib is a worrisome phenomenon that is associated with limited survival benefits and very low response rates [3, 4]. Identifying the molecular mechanisms of sorafenib resistance and improving the response of patients to sorafenib are thus important for the treatment of HCC.

Sorafenib not only blocks the Raf/mitogen-activated protein kinase (MAPK)/extracellular signal-regulated kinase (ERK) signaling pathway but also inhibits a number of tyrosine kinase receptors, including the vascular endothelial growth factor and platelet-derived growth factor receptors [3]. However, sorafenib does not directly target the phosphoinositide 3-kinase (PI3K)/Akt pathway, which is critical for the development and progression of HCC and which is activated in 92.3% of HCC specimens [5, 6]. Sorafenib-induced Akt activation has been reported in both sorafenib-resistant and parental HCC cells [7–9]. Although the mechanisms that underlie the role of Akt activation remain unclear, blocking the PI3K/Akt pathway augments the effects of sorafenib and reverses acquired resistance to sorafenib [7, 9, 10].

Anticancer agents should ultimately induce cell death. Tumor cells that are not killed by chemotherapy are considered drug resistant. Generally, primary resistance is initially due to genetic heterogeneity [4]. However, patients who initially respond to therapy often acquire resistance after long-term exposure to antitumor drugs and will eventually progress [11]. Many mechanisms, such as addiction switching, compensatory pathways due to pathway loops or cross-talk, the epithelial-mesenchymal transition, cancer stem cells, the disabling of pro-apoptotic signals, and a hypoxic microenvironment, are involved in apoptotic pathway imbalance, which leads to cell survival and drug resistance [4, 12, 13]. Moreover, endoplasmic reticulum (ER) stress disturbs the normal functions of the ER via the unfolded protein response (UPR), which has caused widespread concern [14–20]. A number of cellular stress conditions, such as nutrient deprivation, hypoxia, and alterations in glycosylation status, lead to the accumulation of unfolded and/or misfolded proteins in the ER lumen and cause so-called ER stress. The initial physiological aim is to restore ER function by reducing the amount of immature proteins, but this approach can eventually trigger ER-stress-mediated apoptosis if ER dysfunction is severe or prolonged. This approach can also trigger cross-talk with many signaling pathways, including PI3K/Akt [15, 21]. The ER stress pathways consist of three main signaling cascades that are facilitated by the following: inositol-requiring kinase 1 (IRE1), activating transcription factor 6 (ATF6) and protein kinase RNA-like ER kinase [22]. Once activated, protein kinase RNA-like ER kinase phosphorylates eukaryotic initiation factor 2 (eIF2) to inhibit protein translation and upregulate ATF4 and C/EBP-homologous protein (CHOP) expression. ATF6 then translocates into the nucleus and induces the expression of genes that contain an ER stress response element, including CHOP [22, 23]. Accumulating evidence indicates that ER stress is activated in various solid tumors and is involved in drug resistance in many different tumor types, including HCC [14–20]. Moreover, acquired drug resistance is induced by antitumor drugs due to the absence of ER stress [20]. Recently, sorafenib was also reported to induce ER-stress-dependent apoptosis in HCC cells [22, 24].

Bufalin, an ingredient of the traditional Chinese medicine *Chan su*, which is obtained from the skin and parotid venom glands of the toad *Bufo bufo gargarizans Cantor*, has been approved by the Chinese State Food and Drug Administration and is widely used to treat patients with liver, lung, colon and pancreatic cancers at oncology clinics in China [25]. We previously found that bufalin inhibited HCC cell proliferation and induced apoptosis by activating the ER stress response via the IRE1 pathway. We also showed that the molecular mechanism of acquired resistance to sorafenib was Akt activation [7], as inhibition of Akt could enhance antitumor effects [10] and reverse acquired resistance to sorafenib [7]. Importantly, bufalin also suppresses cancer by inhibiting the activation of Akt [26–28]. However, the role of bufalin-induced ER stress and its cross-talk with the PI3K/Akt pathway in mediating HCC resistance to sorafenib has not yet been reported. Herein, we demonstrate that bufalin and sorafenib synergistically act to induce HCC cell death by downregulating phosphorylated (p)-Akt in an ER-stress-dependent manner mediated by the IRE1 pathway. Bufalin also reverses acquired resistance to sorafenib via this mechanism. Therefore, bufalin may help to improve sorafenib treatment by alleviating both inherent and acquired resistance.

## Materials and Methods

### Cell lines and antibodies

Human HCC HepG2 cells were purchased from the American Type Culture Collection (ATCC; Manassas, VA, USA), and Huh7 cells were obtained from the Chinese Academy of Sciences Cell Bank (Shanghai, China). The cells were routinely cultured at 37°C in Dulbecco’s modified Eagle’s medium (DMEM) supplemented with 10% fetal bovine serum. Antibodies (Abs) against Akt, p-Akt (Ser473), CHOP, and p-eIF2α were purchased from Cell Signaling Technology (Danvers, MA, USA), and Abs against IRE1 and β-actin were from Santa Cruz Biotechnology (Santa Cruz, CA, USA). In addition, fluorescein isothiocyanate (FITC)-conjugated anti-rabbit secondary Ab and Alexa Fluor 594-conjugated anti-rabbit and anti-mouse secondary Abs were from Zhongshan Golden Bridge Biotechnology (Beijing, China).

### Reagents

Bufalin and LY294002 were purchased from Sigma (St. Louis, MO, USA). Sorafenib and perifosine were purchased from Jinan Trio Pharmatech Co., Ltd. (Jinan, China). The bufalin, sorafenib and LY294002 were dissolved in 100% dimethyl sulfoxide (DMSO; Sigma, St. Louis, MO, USA) to prepare stock solutions of 1 mM, 100 mM, and 100 mM, respectively, which were diluted in DMEM to the desired concentrations for the *in vitro* assays. All stock solutions contained a final DMSO concentration of less than 0.1%. Perifosine was dissolved in phosphate-buffered saline to prepare a 30 μM stock solution.

### Cell viability assay

Cell viability was analyzed using a Cell Counting Kit-8 (CCK-8) kit (Dojindo Laboratories, Kumamoto, Japan) as described previously [23, 29]. Briefly, the cells were plated in 96-well culture plates at a density of 3×10^3^ cells/well and were cultured overnight. The cells were then incubated in fresh culture medium containing bufalin and/or sorafenib at various concentrations for 24 to 72 h. The cell viability was then assessed according to the manufacturer’s guidelines. The experiments were repeated in triplicate.

### Establishment of sorafenib-resistant cells

The sorafenib-resistant cell lines Huh7-Sora and HepG2-Sora were established by incubating Huh7 and HepG2 cells, respectively, with increasing concentrations of sorafenib, as described previously [7, 30, 31]. The half-maximal inhibitory concentration (IC50) of HCC cells to sorafenib was initially determined, and the cells were then cultured in medium containing sorafenib at concentrations just below their respective IC50 values. The concentration of sorafenib was slowly increased by 0.25 μmol/L per week. After 6 to 7 months, the cells that were able to survive in the medium containing 10 μM sorafenib were considered to be sorafenib resistant.

### Detection of cell apoptosis *in vitro*

Cells were seeded at 5.0×10^5^ cells/well in six-well plates, cultured for 48 h, and then harvested. Apoptosis was detected using an Annexin V-FITC/propidium iodide (PI) apoptosis detection kit (BD Biosciences, Beijing, China). Briefly, the cells were incubated with 5 μl of Annexin V and 5 μl of PI for 15 min. The apoptotic cells were then analyzed by flow cytometry (Beckman Coulter, Brea, CA, USA) or viewed under a laser-scanning confocal microscope (LSM-510 Meta, Carl Zeiss Jena) as described previously [23, 32]. The experiments were repeated in triplicate.

### Caspase activity assay

The activities of caspase-3 and caspase-9 were measured with caspase-3 and caspase-9 activity kits (Beyotime Institute of Biotechnology, Haimen, Jiangsu, China), respectively, according to the manufacturer’s instructions. For this purpose, cells were harvested and lysed, total protein was extracted, and the protein concentration was determined. A mixture of 10 μl of protein extracts, 80 μl of reaction buffer and 10 μl of caspase-3 substrate (Ac-DEVD-pNA) or caspase-9 substrate (Ac-LEHD-pNA) was then incubated in 96-well plates at 37°C for 4 h. The optical density was subsequently measured at 405 nm using a microplate reader. The relative caspase activity was expressed as the percentage of enzyme activity relative to the control, as described previously [10, 32]. The experiments were repeated in triplicate.

### Immunofluorescence assay

Briefly, HCC cells were fixed with 3% paraformaldehyde and permeabilized in 0.1% Triton X-100. The cells were then incubated with the appropriate primary and secondary Abs, and the DNA was stained using DAPI. The immunostained cells were photographed under an inverted fluorescence microscope.

### Immunoblotting

The immunoblotting methodology has been described previously [30, 33]. In brief, protein was extracted, and the protein concentrations of cell lysates were determined using the Bio-Rad Protein Assay (Bio-Rad, Richmond, CA, USA). The lysates were then resolved by SDS/PAGE, and the proteins were transferred to polyvinylidene difluoride (PVDF) membranes. The membranes were blocked with 5% skim milk, incubated with primary Ab and subsequently incubated with alkaline phosphatase-conjugated secondary Ab, followed by detection with enhanced chemiluminescence reagent (Pierce Chemical, Rockford, IL, USA). β-actin was used as a protein loading control, and the protein levels were normalized to the β-actin band density.

### Transfection of siRNAs

The siRNA transfection methods have been previously described in detail [10, 34]. Briefly, cells were grown to 60 to 70% confluence and incubated with siRNAs at a final concentration of 0.1 μM using Lipofectamine^TM^ 2000 (Invitrogen, Beijing, China) in serum-free medium for 24 h. The cells were then subjected to the assays. The siRNAs produced by GenePharma (Shanghai, China) and their targeted genes are shown in Table 1.

**Table 1:** The siRNAs used in this study and their targeted genes.

Gene	GenBank no.	Strands	Reference
Akt	NM_001014431.1	sense strand: 5′-GUGGUCAUGUACGAGAUGATT-3′	[7]
		antisense strand: 5′-UCAUCUCGUACAUGACCACTT-3′	
CHOP	NM_001195053.1	sense strand: 5'-CCAGGAAACGGAAACAGAGTT-3'	[22]
		antisense strand: 5'-CUCUGUUUCCGUUUCCUGGTT-3'	
IRE1	NM_001433.3	sense strand: 5′-GGAAGGUGAUGCACAUCAATT-3′	[22]
		antisense strand: 5′-UUGAUGUGCAUCACCUUCCTC-3′	
eIF2α	NM_032025.3	sense strand: 5′- GGGAAGUACUCAUUAAUAATT-3′	[22]
		antisense strand: 5′- UUAUUAAUGAGUACUUCCCGT-3′	
Control	_	sense strand: 5′-UUCUCCGAACGUGUCACGU-3′	[34]
		antisense strand: 5′-ACGUGACACGUUCGGAGAA-3′	

Abbreviations: IRE1, inositol-requiring enzyme 1; eIF2, eukaryotic initiation factor 2; CHOP, C/EBP-homologous protein.

### Statistical analysis

Quantitative data are expressed as the mean ± standard deviation. Comparisons among multiple groups were performed using one-way analysis of variance (ANOVA) test followed by Dunnett’s test. P<0.05 was considered to indicate significant differences for all analyses.

## Results

### Bufalin synergizes with sorafenib to inhibit HCC cell growth

**Fig 1:**
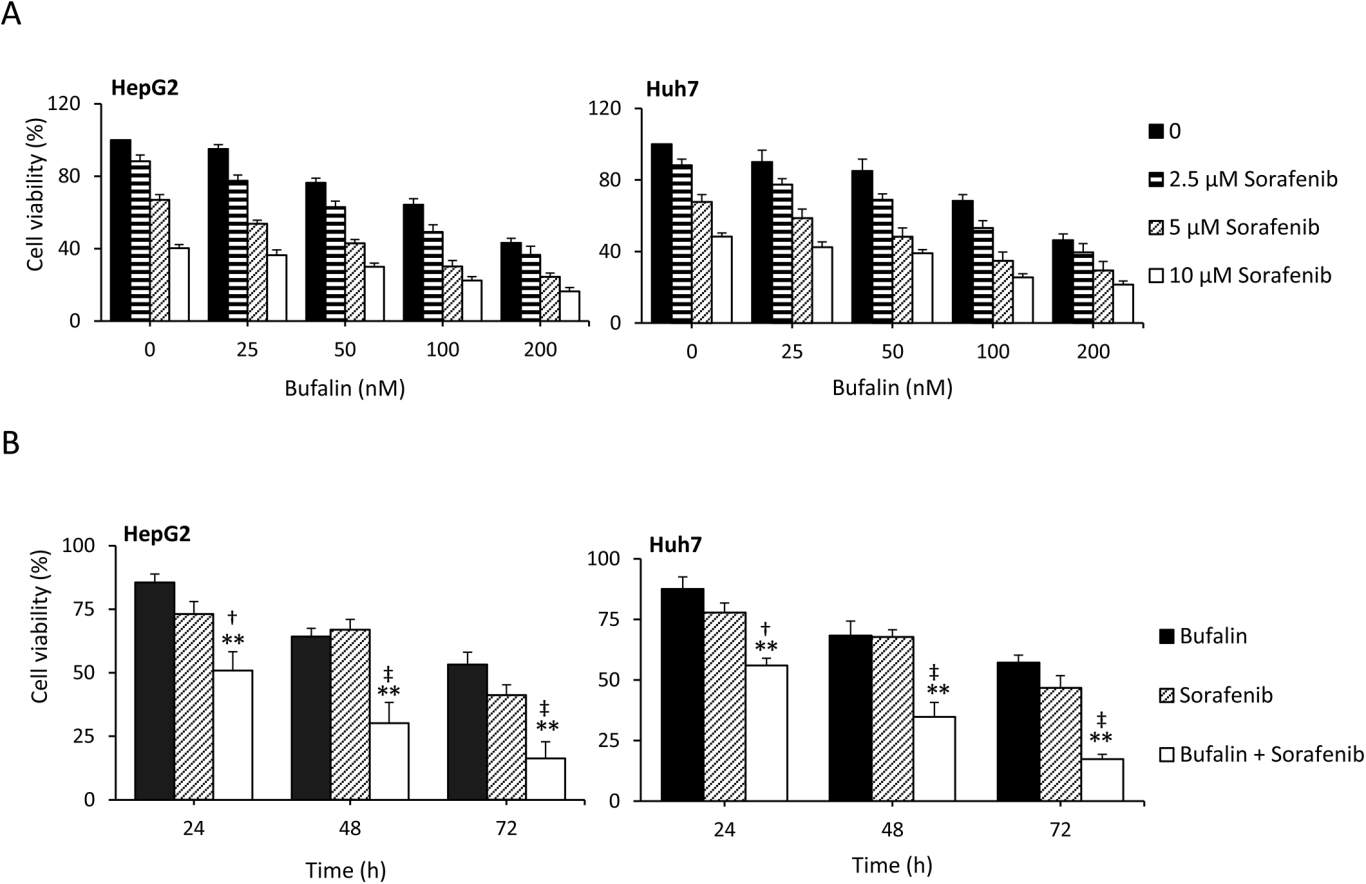
Bufalin synergizes with sorafenib to inhibit HCC cell growth. (A) HepG2 and Huh7 cells were exposed to different concentrations of bufalin and/or sorafenib for 48 h. (B) The above cells were incubated with 100 nM bufalin and/or 5 μM sorafenib for different periods. Cell viability (%) was then compared with the corresponding untreated cells. The data represent three independent experiments. “**” (P<0.001) vs. bufalin alone. “†” (P<0.05) and “‡” (P<0.001) vs. sorafenib alone.

To investigate the effect of bufalin in combination with sorafenib on cell growth, HepG2 and Huh7 cells were incubated with increasing concentrations of bufalin and/or sorafenib for 24, 48, or 72 h. Cell survival was then assessed via the CCK-8 assay. As shown in Fig 1A and 1B, bufalin synergized with sorafenib to inhibit cell viability in a dose- and time-dependent manner. Although 25 nM and 50 nM bufalin alone had a limited effect on the cell growth index, as previously described [23], bufalin in combination with sorafenib significantly reduced cell growth compared with bufalin or sorafenib treatment alone. Moreover, the antitumor effect of bufalin was enhanced at both 100 nM and 200 nM, which demonstrated the dose-dependent activation of bufalin in combination with sorafenib. To investigate whether the effects of bufalin and sorafenib were synergistic, we calculated the coefficient of drug interaction (CDI), as described previously [35–37]. All CDIs for the combination of bufalin and sorafenib at various concentrations were less than 1 (Fig 1A; S1 and S2 Tables), indicating that bufalin and sorafenib synergistically inhibited cell proliferation. Moreover, the combination of 100 nM bufalin and 5 μM sorafenib yielded CDIs of 0.74 and 0.70 for HepG2 and Huh7 cells, respectively. This combination optimized the synergistic effect and was consequently used in subsequent experiments.

### Bufalin synergizes with sorafenib to induce apoptosis in HCC cells

**Fig 2:**
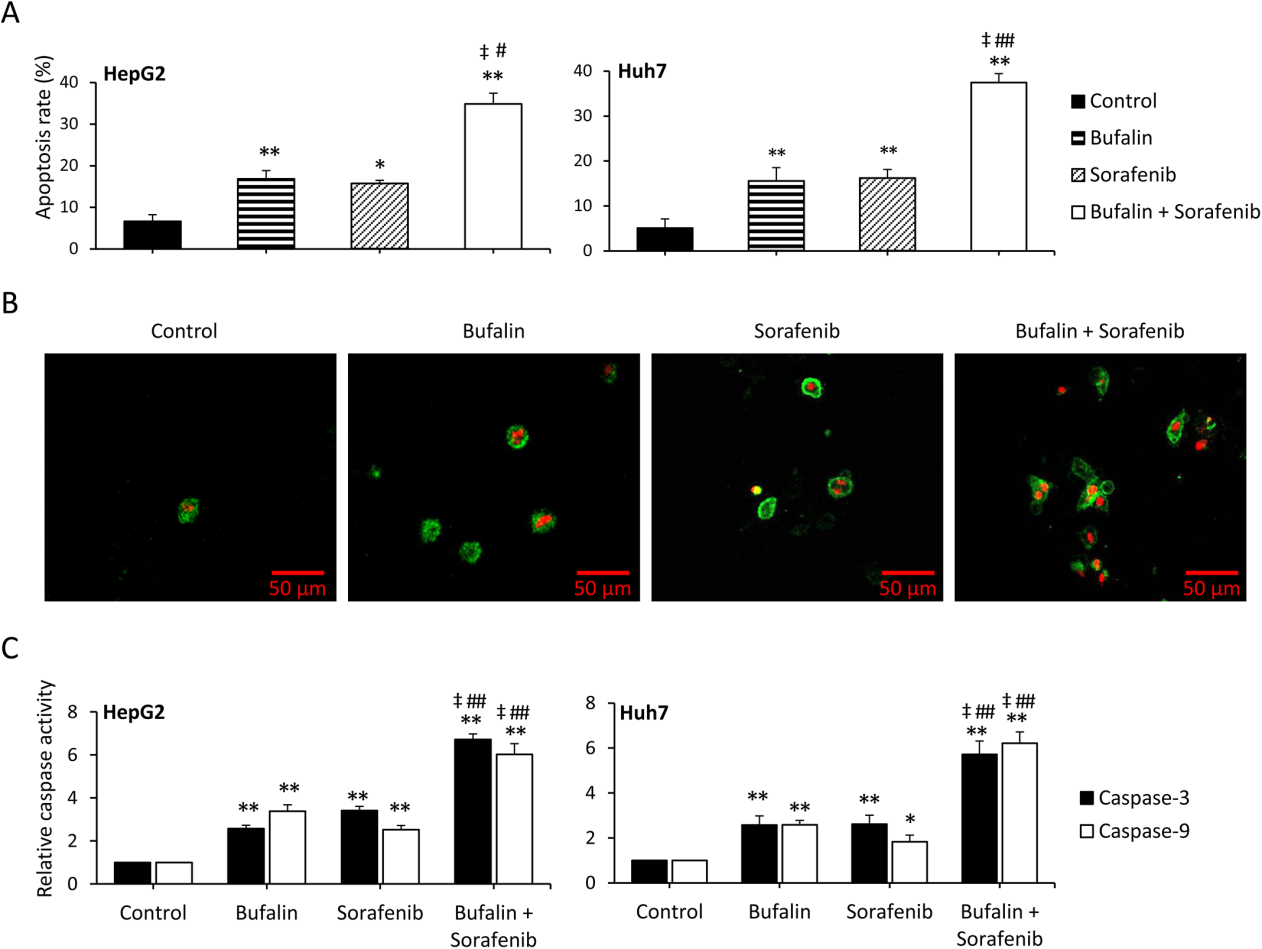
Bufalin synergizes with sorafenib to induce apoptosis in HCC cells. HepG2 and Huh7 cells were incubated with 100 nM bufalin and/or 5 μM sorafenib for 48 h. (A) The cells were stained with Annexin V/PI and subjected to flow cytometry to measure the apoptosis rate (%). (B) Representative images were taken of Huh7 cells stained with Annexin V/PI and viewed with a laser-scanning confocal microscope. (C) The activities of caspase-3 and caspase-9 were measured. The data represent three independent experiments. Untreated cells served as controls. “*” (P<0.05) and “**” (P<0.001) vs. untreated control; “‡” (P<0.001) vs. sorafenib alone; “#” (P<0.05) and “##” (P<0.001) vs. bufalin alone.

To investigate the synergistic effects of bufalin and sorafenib on apoptosis, HepG2 and Huh7 cells were incubated with 100 nM bufalin and/or 5 μM sorafenib for 48 h, harvested, stained with Annexin V/PI, and then subjected to flow cytometry to determine the apoptosis rate. As shown in Fig 2A, the apoptosis rates were 6.6%, 16.8%, 15.7% and 34.9% in HepG2 cells and 4.5%, 13.1%, 12.1%, and 38.7% in Huh7 cells for the control, bufalin, sorafenib and bufalin + sorafenib groups, respectively. Additionally, the CDIs were 0.78 and 0.80 in HepG2 and Huh7 cells, respectively, indicating that bufalin and sorafenib synergistically induced apoptosis. To confirm the ability of bufalin and sorafenib to induce apoptosis, confocal microscopy was used to view the Annexin V/PI-stained cells. As shown in Fig 2B, early-stage apoptotic cells (green fluorescent membranes) and late-stage apoptotic cells (green fluorescent membranes and nuclei) were abundant in bufalin- or sorafenib-treated cells but rare in the untreated control cells. Additionally, treatment with a combination of bufalin and sorafenib increased the number of apoptotic cells compared with bufalin or sorafenib treatment alone. Bufalin and sorafenib treatment each significantly increased the cellular activities of caspase-3 and caspase-9 compared with the activities in untreated cells, and the combination treatment further increased these activities compared with bufalin or sorafenib treatment alone (Fig 2C).

### Bufalin suppresses sorafenib-induced Akt activation to reverse sorafenib resistance in HCC cells

**Fig 3:**
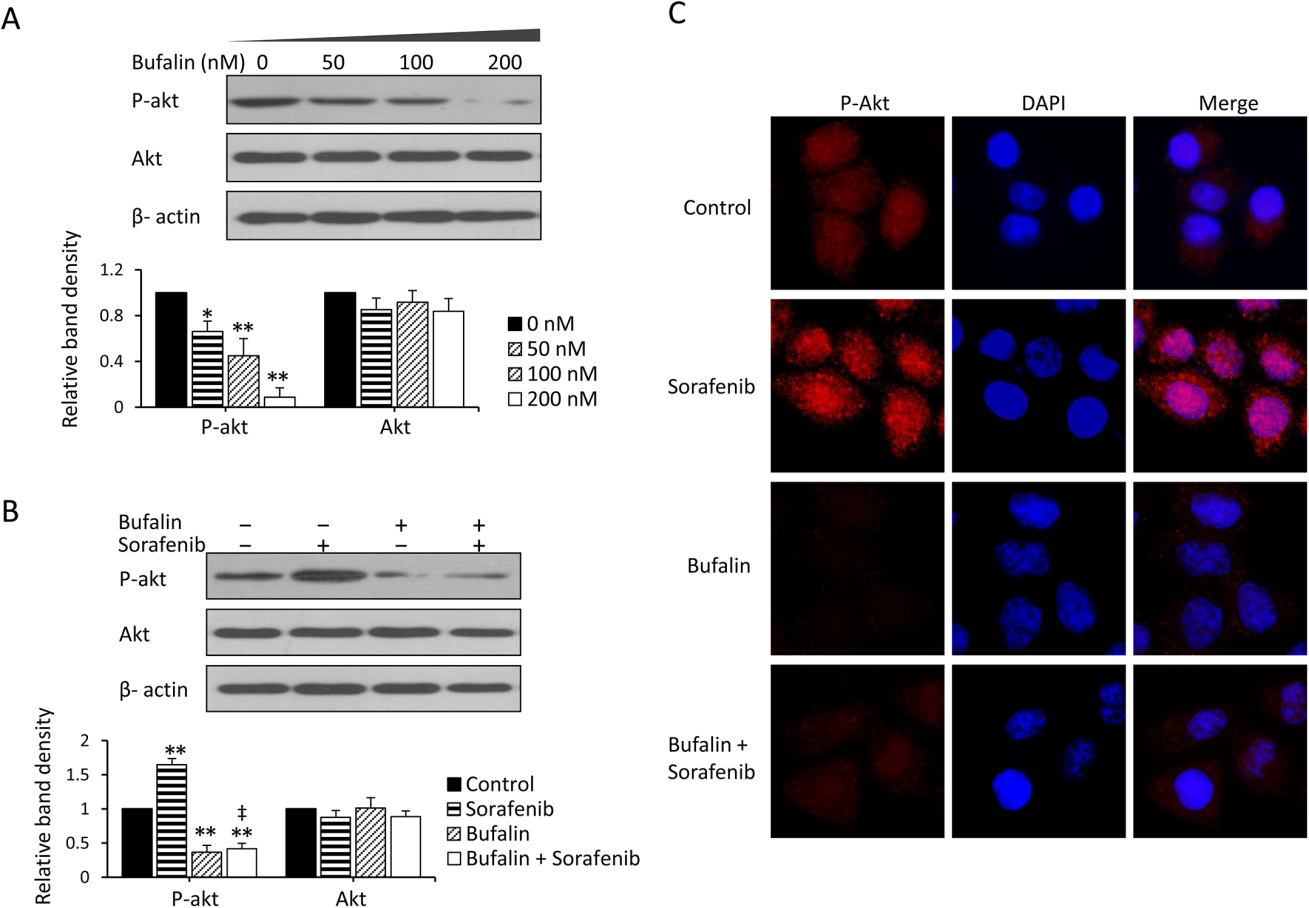
Bufalin suppresses sorafenib-induced Akt activation to reverse resistance to sorafenib in HCC cells. A-B, Huh7 cells were exposed to different concentrations of bufalin (A) or to 100 nM bufalin and/or 5 μM sorafenib (B) for 48 h. Untreated cells served as controls. Cell lysates were immunoblotted, and the density of each band was measured. Band densities were normalized to β-actin. The relative band density from untreated cells was defined as 1. (C) The Huh7 cells from (B) were immunostained with anti-p-Akt Ab (red) and DAPI (cellular nuclei, blue) and viewed with an inverted fluorescence microscope. The data represent three independent experiments. “*” (P<0.05) and “**” (P<0.001) vs. untreated control; “‡” (P<0.001) vs. sorafenib alone.

As shown in our previous study [7, 10], increased p-Akt is responsible for resistance to sorafenib, and specific inhibition of Akt synergizes with sorafenib to inhibit the proliferation and promote the apoptosis of sorafenib-resistant cells both *in vitro* and *in vivo*. Therefore, in the current study, we examined the effect of bufalin on Akt expression. For this purpose, HCC cells were incubated with increasing concentrations of bufalin for 48 h and then subjected to an immunoblotting analysis. Bufalin downregulated the expression of p-Akt in a concentration-dependent manner but did not affect Akt expression (Fig 3A; S1A Fig), and as shown in Fig 3B and S1B Fig, sorafenib upregulated the expression of p-Akt. LY294002, a potent inhibitor of Akt, synergized with sorafenib to inhibit cell viability and promote apoptosis (S2 and S3 Figs), indicating that the sorafenib-induced activation of Akt was responsible for the resistance to sorafenib. When bufalin was combined with sorafenib, bufalin significantly suppressed sorafenib-induced Akt activation (P<0.001; Fig 3B and S1B Fig). The above results were further supported by an immunofluorescence assay (Fig 3C).

**Fig 4:**
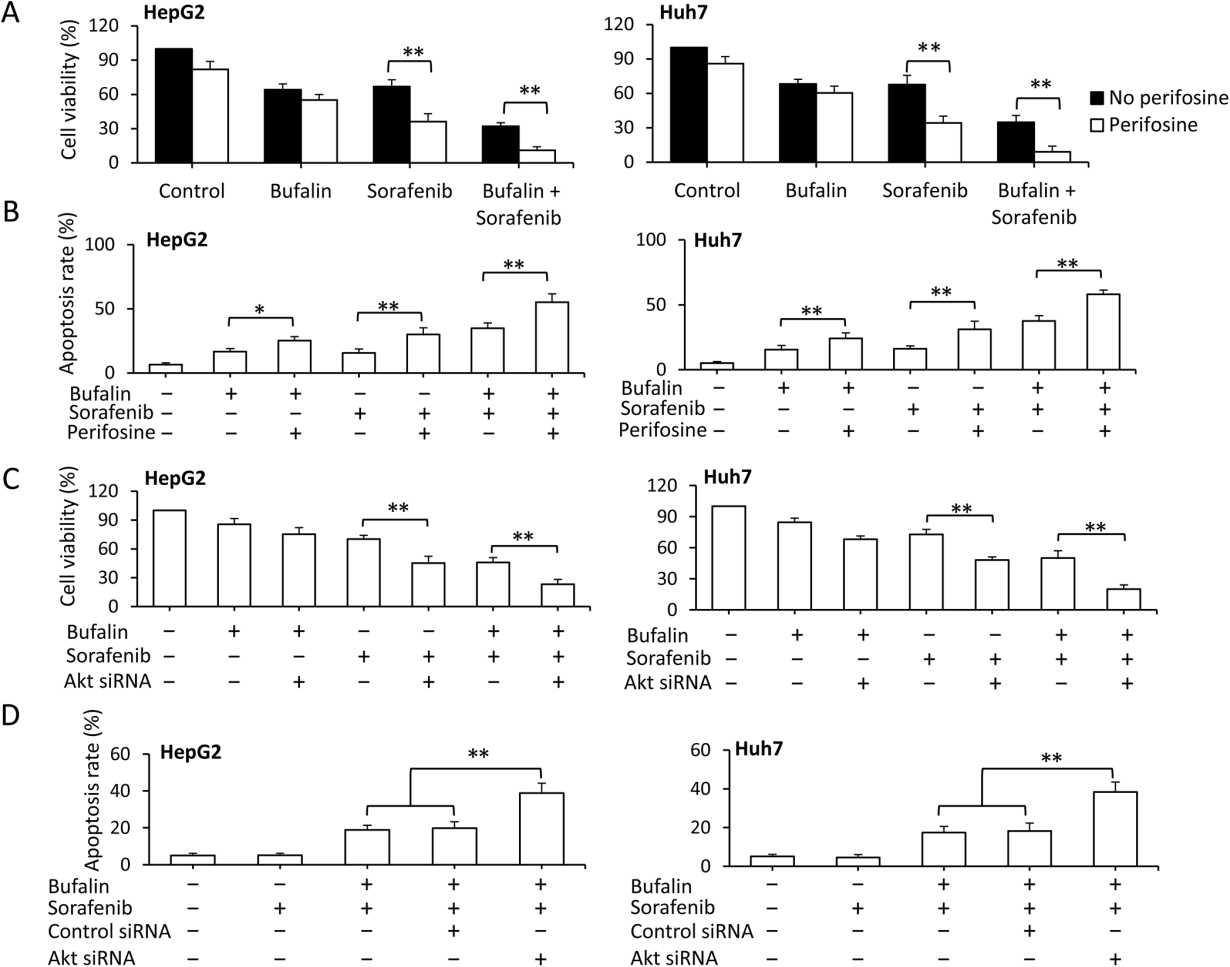
Inhibition of Akt enhances sorafenib-induced growth inhibition and apoptosis. A-B, HepG2 and Huh7 cells were exposed to 100 nM bufalin and/or 5 μM of sorafenib in the presence or absence of perifosine (10 μM) for 48 h. (A) Cell viability (%) was then compared with the corresponding untreated cells. (B) The percentages of apoptotic cells (%) were measured by flow cytometry. Untransfected cells served as controls. C-D, HepG2 and Huh7 cells were transfected with control or Akt siRNA for 24 h and then incubated with 100 nM bufalin, 5 μM sorafenib, or a combination of the two drugs for 24 h. (C) Cell viability (%) was compared with control siRNA-transfected cells. (D) The percentages of apoptotic cells (%) were measured by flow cytometry. Untransfected cells served as controls. The data represent three independent experiments. “**” represents P<0.001.

Consequently, perifosine, another specific Akt inhibitor, was used to determine the role of Akt in the synergistic effects of bufalin and sorafenib. Specifically, HepG2 and Huh7 cells were exposed to 100 nM bufalin and/or 5 μM sorafenib for 48 h in the presence or absence of perifosine. A cell viability analysis showed that perifosine significantly increased the antitumor activity of sorafenib by inhibiting cell growth and inducing apoptosis in both the presence and the absence of bufalin (Fig 4A and 4B). We next examined whether depletion of Akt by siRNAs could potentiate the anticancer activities of bufalin in combination with sorafenib in HCC. HepG2 and Huh7 cells were transfected with control or Akt siRNA for 24 h and then further incubated for 24 h with 100 nM bufalin, 5 μM sorafenib or a combination of these two drugs. As shown in Fig 4C and 4D, the depletion of Akt by siRNA increased the antitumor activity of sorafenib when combined with bufalin by inhibiting cell growth and inducing apoptosis. This finding indicated that bufalin suppresses sorafenib-induced Akt activation, reversing sorafenib resistance in HCC cells.

### Bufalin-induced Akt inactivation is IRE1 dependent

**Fig 5:**
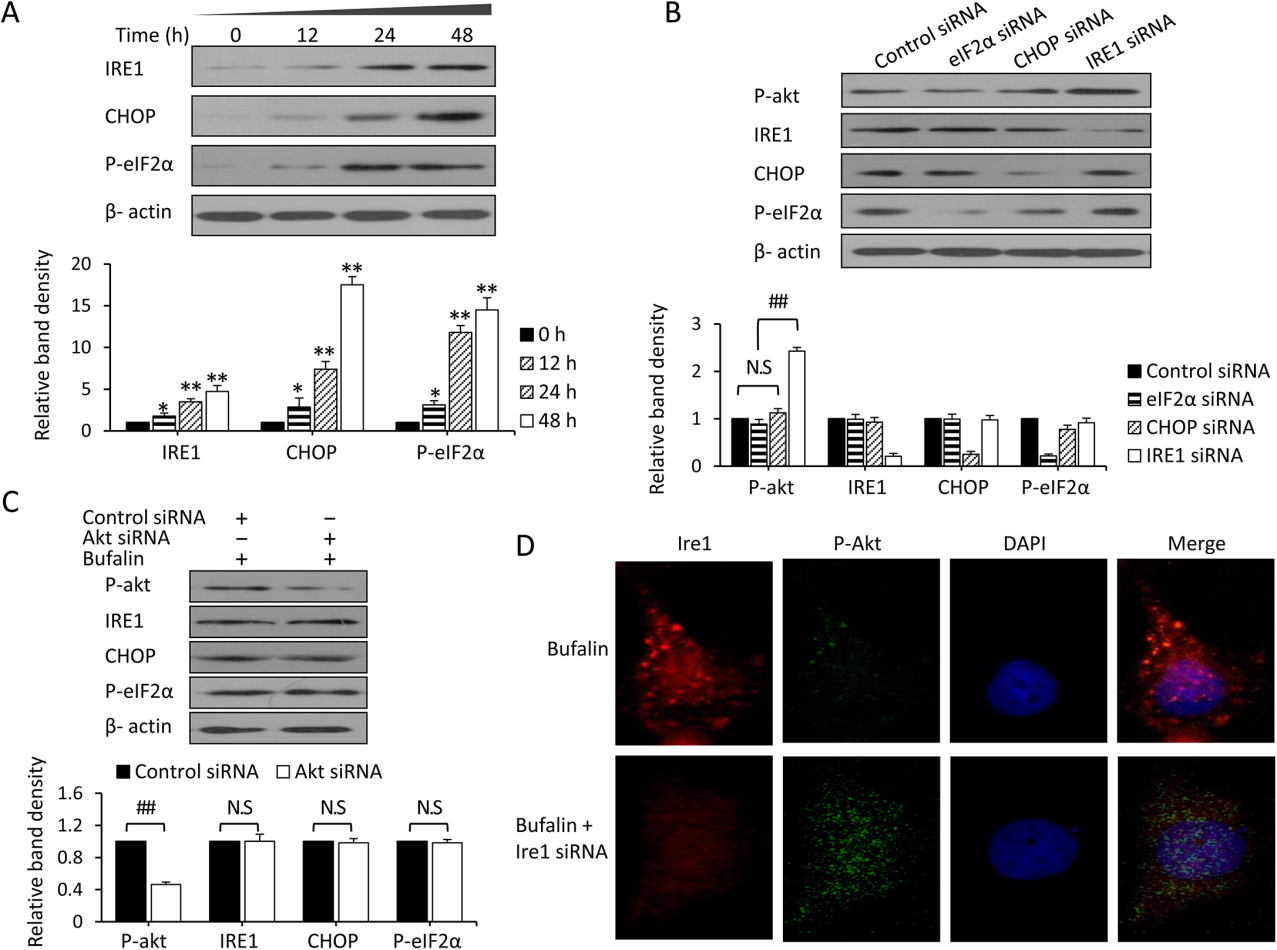
Bufalin-induced Akt inactivation is IRE1 dependent. (A) Huh7 cells were exposed to 100 nM bufalin for 12, 24, or 48 h. Untreated cells served as controls. Cell lysates were immunoblotted, and the density of each band was measured. Band densities were normalized to β-actin. (B) Huh7 cells were transfected with control, eIF2α, CHOP, or IRE1 siRNA for 24 h and then incubated with 100 nM bufalin for 24 h. Cell lysates were immunoblotted, and the density of each band was measured. Band densities were normalized to β-actin. The relative band density from control-siRNA transfected cells was defined as 1. (C) Huh7 cells were transfected with control or Akt siRNA for 24 h and then incubated with 100 nM bufalin for 24 h. Cell lysates were immunoblotted, and the density of each band was measured. Band densities were normalized to β-actin. The relative band density from control-siRNA transfected cells was defined as 1. (D) Huh7 cells were incubated with 100 nM bufalin and/or 5 μM sorafenib for 48 h. The cells were immunostained with Abs against IRE1 (red) and p-Akt (green) as well as with DAPI (cellular nuclei, blue). The data represent three independent experiments. N.S., not significant. “*” (P<0.05) and “**” (P<0.001) vs. untreated control; “##” represents P<0.001.

As shown in our previous study [23], ER stress is involved in the antitumor activities of bufalin. To investigate the role of ER stress in bufalin-induced Akt inactivation, Huh7 cells were incubated with 100 nM bufalin for 0, 12, 24, or 48 h and then subjected to immunoblotting. The protein expression levels of eIF2α, CHOP, and IRE1, the key molecules involved in ER stress, were detected. As shown in Fig 5A, bufalin time-dependently upregulated the expression of eIF2α, CHOP, and IRE1. These results indicate that the three major branches of the UPR are activated in response to ER stress. Moreover, the effect of bufalin on eIF2α, CHOP, and IRE1 expression negatively correlates with p-Akt expression (Fig 3A).

ER stress was recently reported to negatively regulate the AKT/mTOR pathway [38]. We also previously reported that the effects of eIF2α, CHOP, and IRE1 on apoptosis and autophagy are discrepant [23]. Thus, we hypothesized that bufalin-induced Akt inactivation is ER stress dependent. To verify this hypothesis, we used siRNA to knock down the expression of eIF2α, CHOP, or IRE1. In particular, Huh7 cells were transfected with control, eIF2α, CHOP, or IRE1 siRNA for 24 h, followed by further incubation for 24 h with 100 nM bufalin and subsequent immunoblotting. As shown in Fig 5B, the silencing of IRE1 by siRNA reversed the bufalin-induced p-Akt downregulation and significantly increased the expression of p-Akt protein, but the silencing of eIF2α or CHOP did not affect p-Akt expression. Next, Akt siRNA was used to silence Akt, and eIF2α, CHOP, and IRE1 expression was detected by immunoblotting. As shown in Fig 5C, the silencing of Akt by siRNA significantly downregulated the expression of p-Akt protein but did not affect eIF2α, CHOP, or IRE1 expression. Moreover, silencing IRE1 with siRNA in HepG2 cells reversed bufalin-induced p-Akt downregulation, but Akt siRNA did not affect IRE1 expression (S4 Fig). The immunofluorescence staining results revealed that silencing IRE1 with siRNA reversed the bufalin-induced p-Akt downregulation (Fig 5D). When IRE1 was depleted by IRE1 siRNA, inhibition of proliferation and stimulation of apoptosis by bufalin alone or in combination with sorafenib were blocked (S5 Fig). These results indicate that bufalin-induced p-Akt downregulation is related to ER stress and depends on IRE1.

### Bufalin reverses acquired resistance to sorafenib by downregulating p-Akt via IRE1 activation

**Fig 6:**
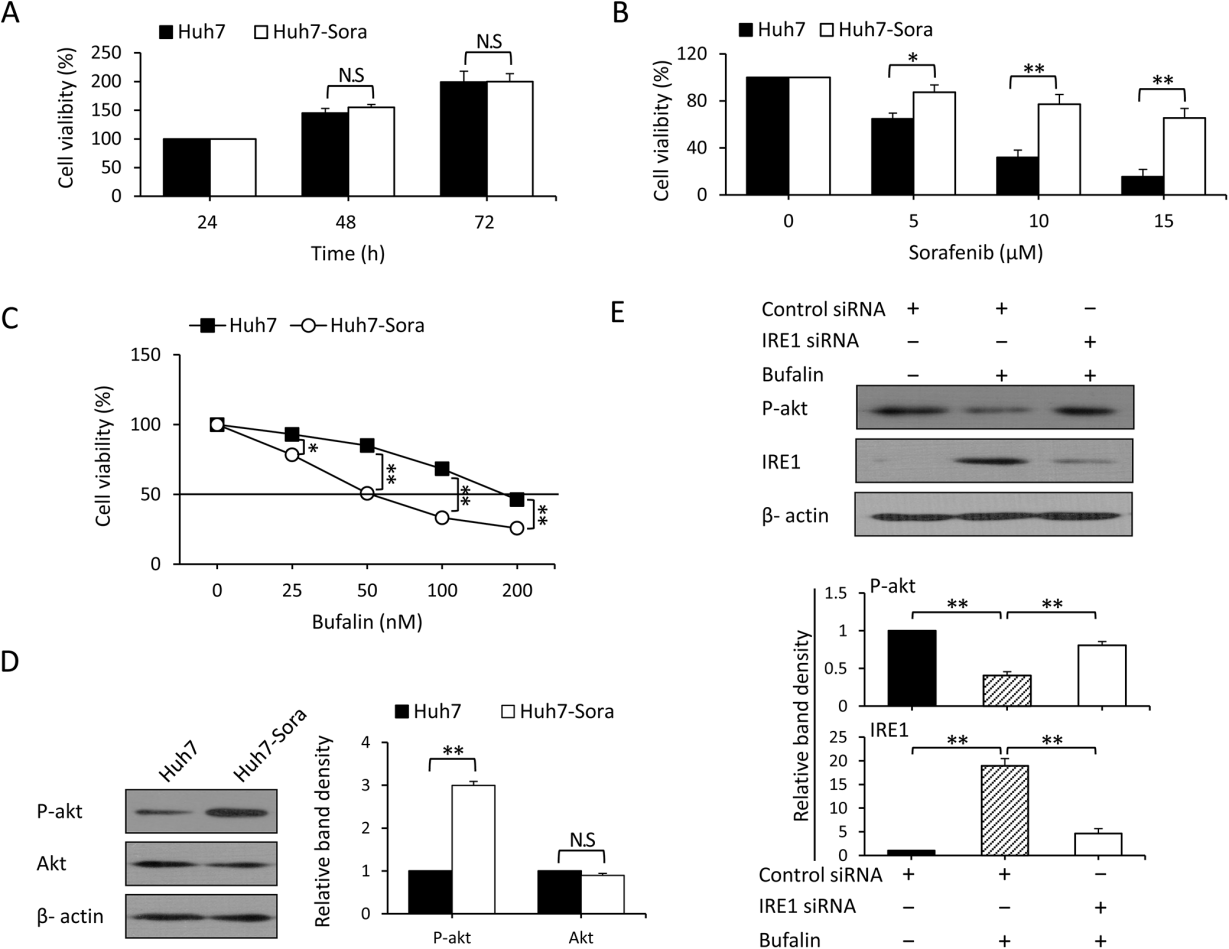
Bufalin reverses acquired resistance to sorafenib by downregulating p-Akt via IRE1 activation. (A) Huh7 and Huh7-Sora cells were cultured in complete medium, and the viability was examined after 24, 48, and 72 h in culture. (B) The above cells were exposed to increasing concentrations of sorafenib for 48 h. Untreated cells served as controls. Cell viability (%) was compared to the corresponding untreated cells. (C) The above cells were exposed to increasing concentrations of bufalin for 48 h. Untreated cells served as controls. Cell viability (%) was compared with the corresponding untreated cells. The black line indicates the IC50. (D) The lysates of cells from (A) were subjected to immunoblotting. Band densities were normalized to β-actin. The relative band density from Huh7 cells was defined as 1. (E) Huh7-Sora cells were transfected with control or IRE1 siRNA for 24 h and then incubated with 100 nM bufalin for 24 h. Cell lysates were immunoblotted, and the density of each band was measured. Band densities were normalized to β-actin. The relative band density from control cells was defined as 1. The data represent three independent experiments. N.S., not significant. “*” represents P<0.05, “**” represents P<0.001.

To examine the role of bufalin in reversing acquired resistance to sorafenib, we continuously exposed Huh7 and HepG2 cells to gradually increasing concentrations of sorafenib. After several months of culture, sorafenib-resistant Huh7-Sora and HepG2-Sora cells were established. As showed in Fig 6A, Huh7-Sora recovered a proliferative capacity comparable to that of the corresponding parental cells. To investigate the sorafenib-resistant characteristics of Huh7-Sora cells, the Huh7-Sora and corresponding parental cells were exposed to increasing concentrations of sorafenib for 48 h, and the cell viability was then detected. As shown in Fig 6B, the cell viability of Huh7-Sora cells was significantly higher than that of Huh7 cells. Even at 15 mmol/L sorafenib, the viability of Huh7-Sora remained at 65.6%, whereas most Huh7 cells had died (15.6%). This finding indicates that Huh7-Sora cells were less sensitive to sorafenib than their parental counterparts were. We then examined the antitumor activity of bufalin in sorafenib-resistant cells. We observed that Huh7-Sora cells were more sensitive to the growth-inhibitory effects of bufalin than their control counterparts were (IC50 55.85±0.6 nM vs. 179.75±1.3 nM; Fig 6C). The HepG2-Sora cells also recovered their proliferative capacity and showed decreased sensitivity to sorafenib, accompanied by an increased sensitivity to bufalin that was comparable to that of the parental cells (S6 Fig).

Based on the above results, we investigated whether the ability of bufalin to activate ER stress and inhibit Akt activation was responsible for the increased sensitivity to bufalin in the sorafenib-resistant cells. We first detected the protein expression of Akt in Huh7 and Huh7-Sora cells. As shown in Fig 6D, p-Akt was significantly upregulated in Huh7-Sora cells compared with Huh7 cells, whereas the difference in Akt expression was not significant between these two cell types. We then incubated control siRNA- or IRE1 siRNA-transfected Huh7-Sora cells with sorafenib for 24 h and examined p-Akt and IRE1 expression. Bufalin upregulated the expression of IRE1 and downregulated p-Akt in control siRNA-transfected cells (Fig 6E). However, when IRE1 was depleted by IRE1 siRNA, bufalin-induced p-Akt downregulation was blocked in Huh7-Sora cells, and p-Akt was upregulated (Fig 6E). The silencing of IRE1 by siRNA inhibited the antitumor activity of bufalin both alone and in combination with sorafenib due to pro-proliferation and anti-apoptosis effects (S7 Fig). These results indicate that bufalin reverses acquired resistance to sorafenib by downregulating p-Akt via IRE1 activation.

## Discussion

Although sorafenib represents the standard first-line systemic treatment for advanced HCC, drug resistance to sorafenib, which is characterized by a low partial response rate to sorafenib, is a unique concern due to the shortage of alternative systemic treatments for HCC [4, 39]. The present study has demonstrated that bufalin, an ingredient of the traditional Chinese medicine *Chan su* that has been approved to treat tumor patients, synergizes with sorafenib to suppress the growth of HCC cells both *in vitro* and *in vivo*. The synergistic effect of these two agents is primarily due to the ability of bufalin to mitigate the sorafenib-induced activation of Akt, which contributes to the resistance of HCC cells to sorafenib. Furthermore, both of these drugs can individually inhibit the proliferation of HCC cells and induce apoptosis. Moreover, the bufalin-induced p-Akt downregulation depends on ER stress and is mediated by the IRE1 pathway. This mechanism is also involved in the ability of bufalin to reverse sorafenib resistance. The encouraging results presented herein warrant future investigation of the use of bufalin as an HCC treatment, especially in combination with sorafenib.

The PI3K/Akt pathway is involved in the development and progression of HCC and is activated in 92.3% of HCC specimens [5, 6]. Therefore, this pathway is a key target of HCC treatment. Sorafenib was previously reported to activate the PI3K/Akt pathway, and blocking the PI3K/Akt signaling pathway enhances the efficacy of sorafenib [8, 10, 40, 41]. In a previous report, we showed that sustained sorafenib treatment could activate Akt in both human HCC patients and mouse HCC models [7]. Here, we showed that sorafenib activated Akt in both parental and sorafenib-resistant HCC cells. The PI3K/Akt pathway includes over one hundred molecules and interacts with tumor-associated pathways, such as those involved in cell growth, invasion, metastasis, apoptosis, and autophagy, among other pathways [7]. Therefore, in the present study, blocking Akt with a specific inhibitor, such as siRNA or bufalin, in combination with sorafenib synergistically inhibited cell growth and induced apoptosis by deregulating downstream factors in HCC cells to reverse both inherent and acquired resistance to sorafenib. This finding is in agreement with the results of our previous report [7, 10].

ER stress is a cellular stress response mediated by the UPR, which is activated by different stimuli, including chemotherapy drugs. The effect of ER stress on tumor cells is pleiotropic and involves pro-survival or pro-apoptotic signals [21–23, 42]. When ER stress is extensive or sustained, the function of the ER cannot be restored, leading to caspase-dependent cell death [43]. Therefore, ER stress is involved in the antitumor activities of numerous chemotherapy drugs for HCC [16, 22], breast cancer [18], ovarian cancer [17], and nasopharyngeal tumors [44], among other cancers. However, the role of ER stress in chemotherapy resistance in HCC [16], breast cancer [18, 45], and ovarian cancer [17] remains controversial. Certain research has shown that ER stress induces drug resistance and that inhibition of ER stress reverses drug resistance [14–16]. Other research has shown that ER stress is responsible for the sensitivity of cancer cells to chemotherapy drugs [45] and that inducing ER-stress-associated apoptosis reverses drug resistance [46, 47]. Moreover, the inability of anticancer drugs to induce ER stress is responsible for acquired resistance to chemotherapy in human laryngeal carcinoma cells [20]. Sorafenib-induced ER-stress-related apoptosis has also been reported in HCC [22, 24]. Here, we showed that bufalin-induced ER stress activation synergistically enhanced the antitumor activity of sorafenib in parental cells and reversed acquired sorafenib resistance in sorafenib-resistant HCC cells. However, we did not investigate the effect of sorafenib on ER stress in the drug resistance model. Nevertheless, the above results at least partly support the idea that ER stress may also be involved in acquired drug resistance to sorafenib because sustained drug exposure could induce an adaptive response consisting of downregulation of sorafenib-induced ER stress, inhibition of apoptosis pathways and activation of compensatory pro-survival signals. Generally, ER-stress-induced apoptosis depends on caspase-4 and caspase-9, but the roles of caspase-3 and caspase-9 have not yet been reported [23, 48]. Cross-talk between ER stress signals and cell death signals, such as PI3K/Akt, Raf/MAPK/ERK, and autophagy, has also been observed [22, 23, 49]. ER stress was recently reported to negatively regulate p-Akt expression [38] and to activate Akt [15] as well. One report also showed that PI3K/Akt inactivation could mediate ER-stress-induced ERK activation [43]. Therefore, the complex effect of ER stress on the PI3K/Akt pathway in sorafenib resistance remains unclear. Here, we showed that bufalin downregulated p-Akt in parental and sorafenib-resistant HCC cells in an ER-stress-dependent manner. However, the roles of the three main downstream effectors of ER stress in the induction of cell death differ [22, 38], which is in agreement with our previous report [23]. To investigate whether the roles of IRE1, eIF2α, and ATF6 are different, IRE1 and eIF2α were directly knocked out with IRE1 and eIF2α siRNAs, respectively, and siRNA was also used to block CHOP, the downstream cofactor of eIF2α and ATF6. Our novel results indicate that ER-stress-induced p-Akt inactivation is IRE1 dependent but independent of eIF2α and ATF6.

Bufalin, the digoxin-like primary component of the traditional Chinese medicine *Chan su*, was initially identified more than 1000 years ago in extracts from the skin and parotid venom glands of *Bufo bufo gargarizans Cantor*. This drug has a broad spectrum of biological activities, including cardiotonic, anesthetic, blood pressure stimulatory, and respiratory effects [26, 50]. In modern Chinese medical practice, the drug has been widely used in clinical trials to treat various intermediate and advanced solid tumors due to its antitumor activity [23, 25, 49, 51, 52]. Recently, bufalin has garnered increasing attention due to its ability to inhibit cell growth and induce apoptosis in HCC cells and animal models of HCC, a cancer that is notoriously inherently resistant to systemic chemotherapy [26–28, 51, 53, 54]. However, the antitumor mechanism of bufalin is complex and controversial. Bufalin has been reported to increase the expression of p-Akt in HCC cells at concentrations of 50 nM and 250 nM [49]. However, at the routine concentration of 100 nM [26–28] and at the low level of 10 nM [53], bufalin consistently downregulated p-Akt expression. Here, we have demonstrated that bufalin dose-dependently downregulated p-Akt expression, which is in accordance with previous reports [26, 27]. Recently, bufalin was also reported to reverse resistance to 5-fluorouracil and doxorubicin in HCC [52, 55]. Moreover, bufalin could enhance the anti-proliferative effects of sorafenib in HCC cells [49]. The antitumor activity of bufalin has been attributed to its ability to induce ER stress [56], which is in agreement with our previous report [23]. Here, we specifically reported that bufalin-induced p-Akt downregulation was ER stress dependent and mediated by the IRE1 pathway. Bufalin also reversed acquired resistance to sorafenib via this pathway.

In summary, the present study demonstrated that bufalin reversed both inherent and acquired resistance to sorafenib in HCC cells by suppressing sorafenib-mediated Akt activation. This effect may be due to bufalin-induced ER stress, which subsequently downregulated p-Akt, inhibited cell growth and induced apoptosis. Moreover, this activity depended on the IRE1 pathway. Although the latent mechanism by which bufalin reverses resistance to sorafenib is complex and unclear, the ER stress pathway engages in cross-talk with many pathways related to cell growth and apoptosis. We showed that the bufalin-induced inactivation of Akt at least partly depended on ER stress and was mediated by the IRE1 pathway. These results warrant further studies of the utility of bufalin alone or in combination with sorafenib as a first- or second-line treatment after sorafenib failure for advanced HCC.

## Supporting Information

S1 Fig Bufalin suppresses sorafenib-induced Akt activation in HCC cells. A-B, HepG2 cells were exposed to increasing concentrations of bufalin (A) or to 100 nM bufalin and/or 5 μM sorafenib (B) for 48 h and then subjected to immunoblotting. Band densities were normalized to β-actin. The relative band density from untreated cells was defined as 1. The data represent three independent experiments. “**” (P<0.001) vs. untreated control; “‡” (P<0.001) vs. sorafenib alone; “#” (P<0.05) and “##” (P<0.001) vs. bufalin alone.
(TIF)

S2 Fig Inhibition of Akt enhances sorafenib-induced growth inhibition. HepG2 (A) and Huh7 (B) cells were incubated with 10 mM LY294002, 5 μM sorafenib, or a combination of the two drugs for 48 h. Untransfected cells served as controls. Cell viability (%) was compared with the corresponding untreated cells. The data represent three independent experiments. “*” (P<0.05) and “**” (P<0.001) vs. untreated control; “‡” (P<0.001) vs. sorafenib alone; “##” (P<0.001) vs. LY294002 alone.
(TIF)

S3 Fig Inhibition of Akt enhances sorafenib-induced apoptosis. HepG2 (A) and Huh7 (B) cells were incubated with 10 mM LY294002, 5 μM sorafenib, or a combination of the two drugs for 48 h. Untransfected cells served as controls. The cells were analyzed using flow cytometry to detect apoptosis. The data represent three independent experiments. “*” (P<0.05) and “**” (P<0.001) vs. untreated control; “‡” (P<0.001) vs. sorafenib alone; “##” (P<0.001) vs. LY294002 alone.
(TIF)

S4 Fig Bufalin-induced Akt inactivation is IRE1 dependent. A-B, HepG2 cells were transfected with control, Akt or IRE1 siRNA for 24 h and then incubated with or without 100 nM bufalin for 24 h. Cell lysates were immunoblotted, and representative bands are shown (A). (B) The density of each band in (A) was measured and normalized to β-actin. The data represent three independent experiments. N.S., not significant. “**” represents P<0.001.
(TIF)

S5 Fig Silencing of IRE1 by siRNA inhibits the antitumor activity of bufalin in Huh7 cells. A-B, Huh7 cells were transfected with control or IRE1 siRNA for 24 h and then incubated with 100 nM bufalin, 5 μM sorafenib, or a combination of the two drugs for 48 h. (A) Cell viability (%) was measured. (B) The percentages of apoptotic cells (%) were measured by flow cytometry. Control siRNA-transfected cells served as controls. The relative band density from control cells was defined as 1. The data represent three independent experiments. “*” represents P<0.05, “**” represents P<0.001.
(TIF)

S6 Fig Sorafenib-resistant HCC cells display increased sensitivity to bufalin. (A) HepG2 and HepG2-Sora cells were cultured in complete medium, and the viability was examined after 24, 48, and 72 h in culture. (B) The above cells were exposed to increasing concentrations of sorafenib for 48 h. Untreated cells served as controls. Cell viability (%) was compared with the corresponding untreated cells. (C) The above cells were exposed to increasing concentrations of bufalin for 48 h. Untreated cells served as controls. Cell viability (%) was compared with to the corresponding untreated cells. The data represent three independent experiments. N.S., not significant. The black line indicates the IC50. “*” represents P<0.05, “**” represents P<0.001.
(TIF)

S7 Fig Silencing of IRE1 by siRNA inhibits the antitumor activity of bufalin in Huh7-Sora cells. A-B, Huh7-Sora cells were transfected with control or IRE1 siRNA for 24 h and then incubated with 50 nM bufalin, 10 μM sorafenib, or a combination of the two drugs for 48 h. (A) Cell viability (%) was measured. (B) The percentages of apoptotic cells (%) were measured by flow cytometry. Control siRNA-transfected cells served as controls. The data represent three independent experiments. “**” represents P<0.001.
(TIF)

S1 Table The CDIs of bufalin in combination with sorafenib in HepG2 cells. (DOCX)

S2 Table The CDIs of bufalin in combination with sorafenib in Huh7 cells. (DOCX)

## Abbreviations

HCC: hepatocellular carcinoma
MAPK: mitogen-activated protein kinase
ERK: extracellular signal-regulated kinase
PI3K: phosphatidylinositol 3-kinase
ER: endoplasmic reticulum
UPR: unfolded protein response
IRE1: inositol-requiring enzyme 1
ATF6: transcription actor 6
eIF2: eukaryotic initiation factor 2
CHOP: C/EBP-homologous protein
DMEM: Dulbecco’s modified Eagle’s medium
Abs: antibodies
FITC: fluorescein isothiocyanate
DMSO: dimethyl sulfoxide
CCK-8: Cell Counting Kit-8
IC50: half-maximal inhibitory concentration
PI: propidium iodide
PVDF: polyvinylidene difluoride
ANOVA: one-way analysis of variance
CDI: coefficient of drug interaction
