# Supplementary figures and images for: Bufalin Reverses Resistance to Sorafenib by Inhibiting Akt Activation in Hepatocellular Carcinoma: The Role of Endoplasmic Reticulum Stress
Source: PLoS One. 2015 Sep 18;10(9):e0138485. doi: 10.1371/journal.pone.0138485 (PMC4575108; doi:10.1371/journal.pone.0138485)

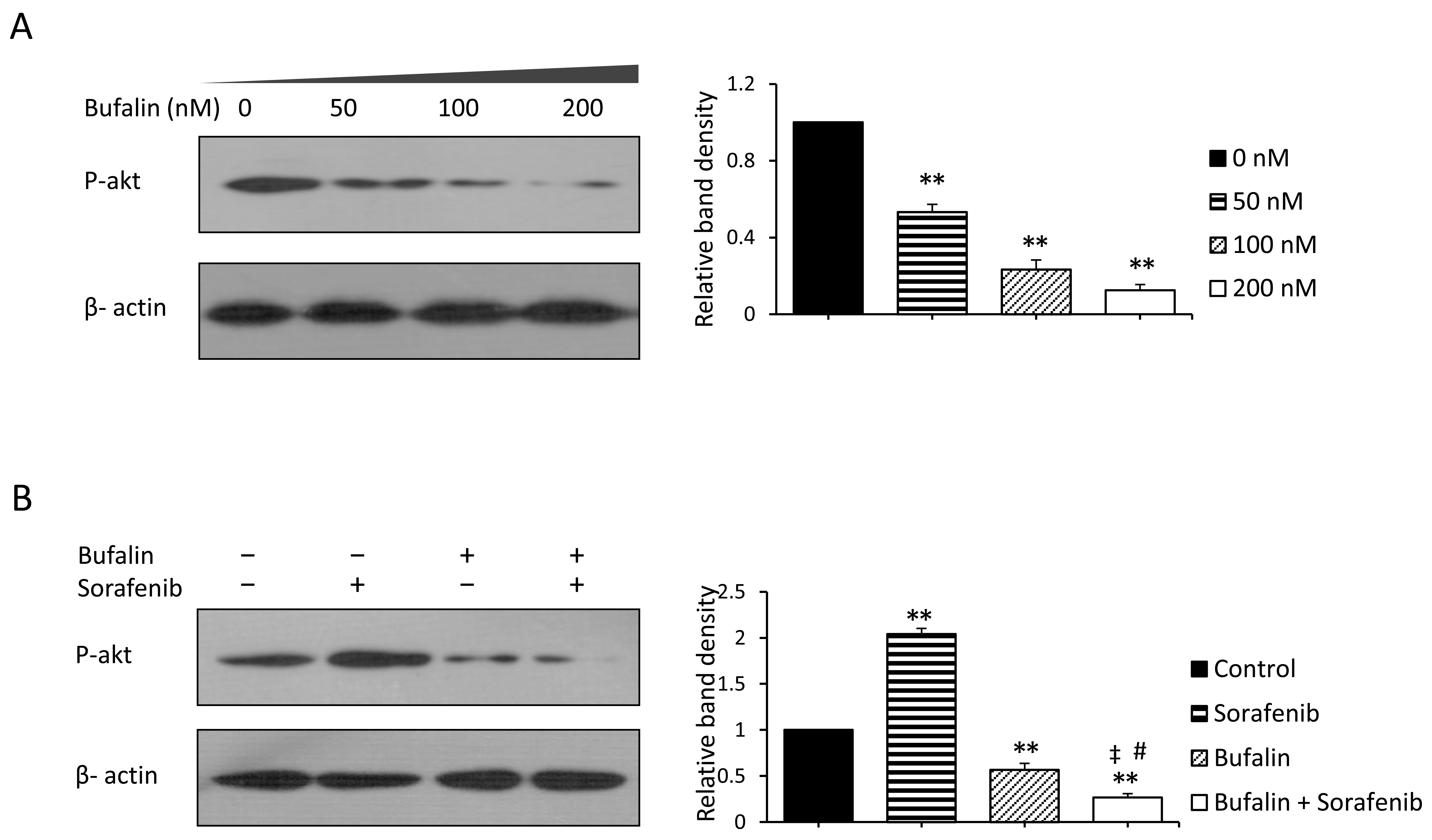

Supplement: S1 Fig — A-B, HepG2 cells were exposed to increasing concentrations of bufalin (A) or to 100 nM bufalin and/or 5 μM sorafenib (B) for 48 h and then subjected to immunoblotting. Band densities were normalized to β-actin. The relative band density from untreated cells was defined as 1. The data represent three independent experiments. “**” (P<0.001) vs. untreated control; “‡” (P<0.001) vs. sorafenib alone; “#” (P<0.05) and “##” (P<0.001) vs. bufalin alone. (TIF) [file pone.0138485.s001.tif]

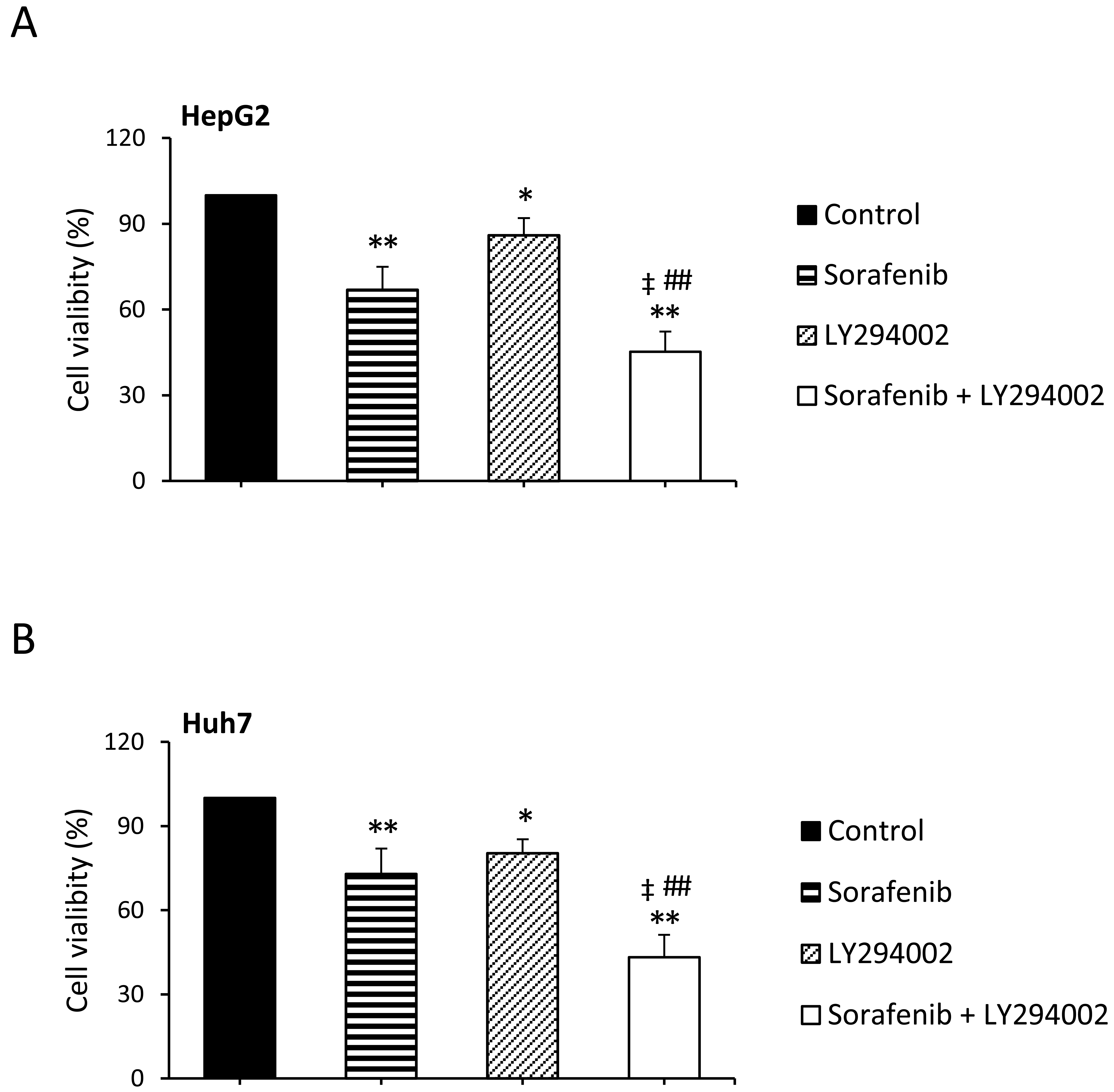

Supplement: S2 Fig — HepG2 (A) and Huh7 (B) cells were incubated with 10 mM LY294002, 5 μM sorafenib, or a combination of the two drugs for 48 h. Untransfected cells served as controls. Cell viability (%) was compared with the corresponding untreated cells. The data represent three independent experiments. “*” (P<0.05) and “**” (P<0.001) vs. untreated control; “‡” (P<0.001) vs. sorafenib alone; “##” (P<0.001) vs. LY294002 alone. (TIF) [file pone.0138485.s002.tif]

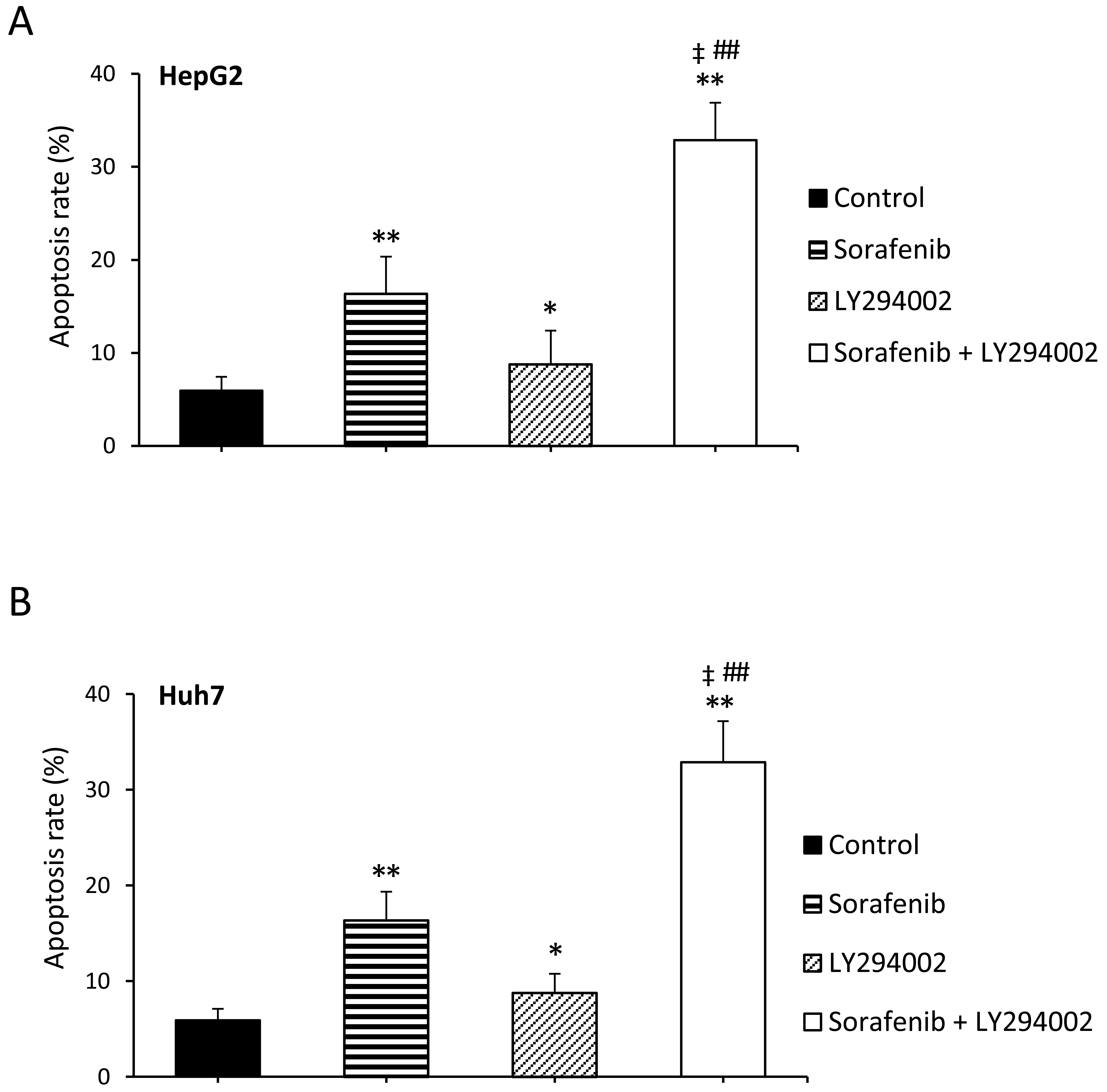

Supplement: S3 Fig — HepG2 (A) and Huh7 (B) cells were incubated with 10 mM LY294002, 5 μM sorafenib, or a combination of the two drugs for 48 h. Untransfected cells served as controls. The cells were analyzed using flow cytometry to detect apoptosis. The data represent three independent experiments. “*” (P<0.05) and “**” (P<0.001) vs. untreated control; “‡” (P<0.001) vs. sorafenib alone; “##” (P<0.001) vs. LY294002 alone. (TIF) [file pone.0138485.s003.tif]

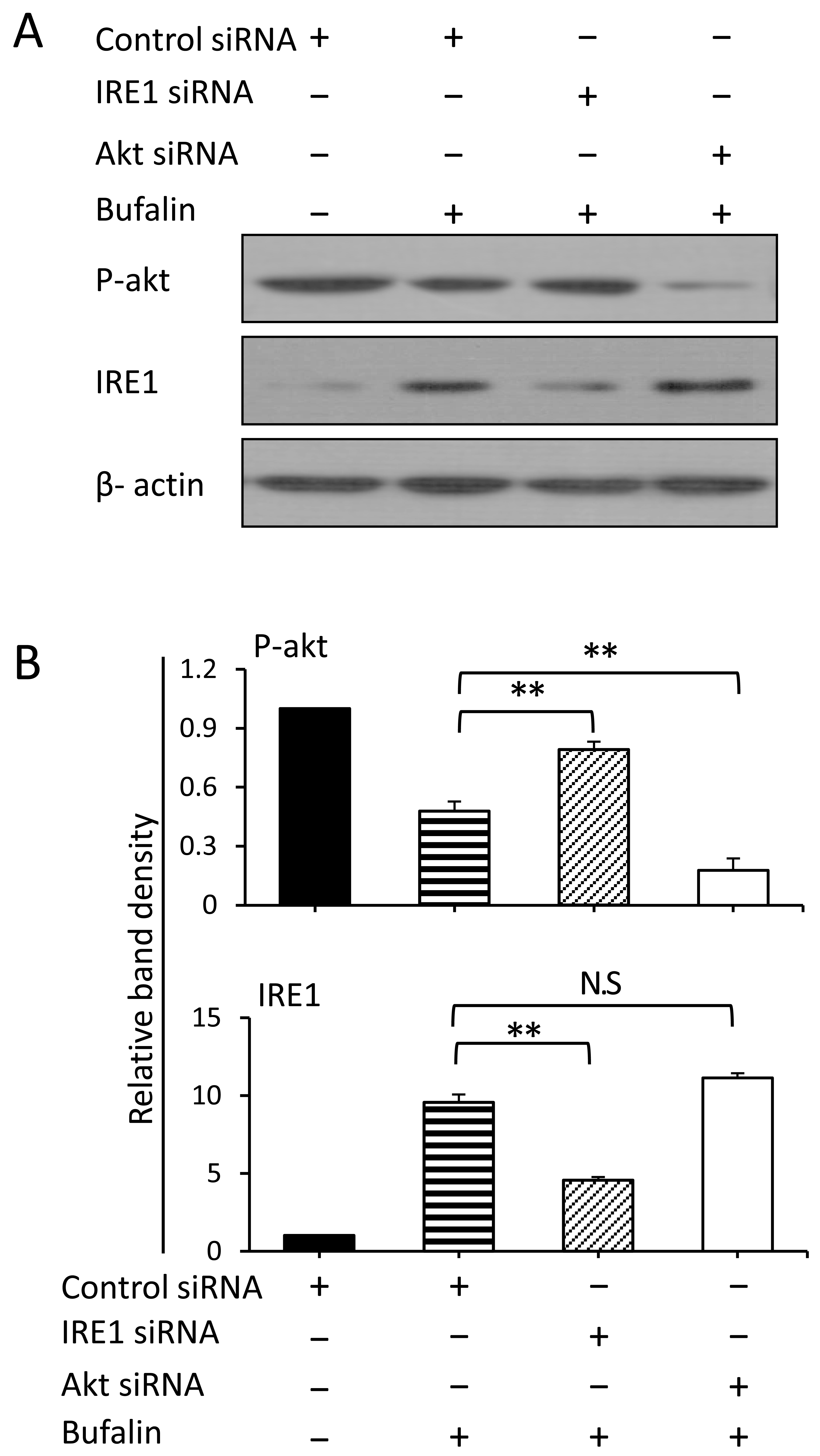

Supplement: S4 Fig — A-B, HepG2 cells were transfected with control, Akt or IRE1 siRNA for 24 h and then incubated with or without 100 nM bufalin for 24 h. Cell lysates were immunoblotted, and representative bands are shown (A). (B) The density of each band in (A) was measured and normalized to β-actin. The data represent three independent experiments. N.S., not significant. “**” represents P<0.001. (TIF) [file pone.0138485.s004.tif]

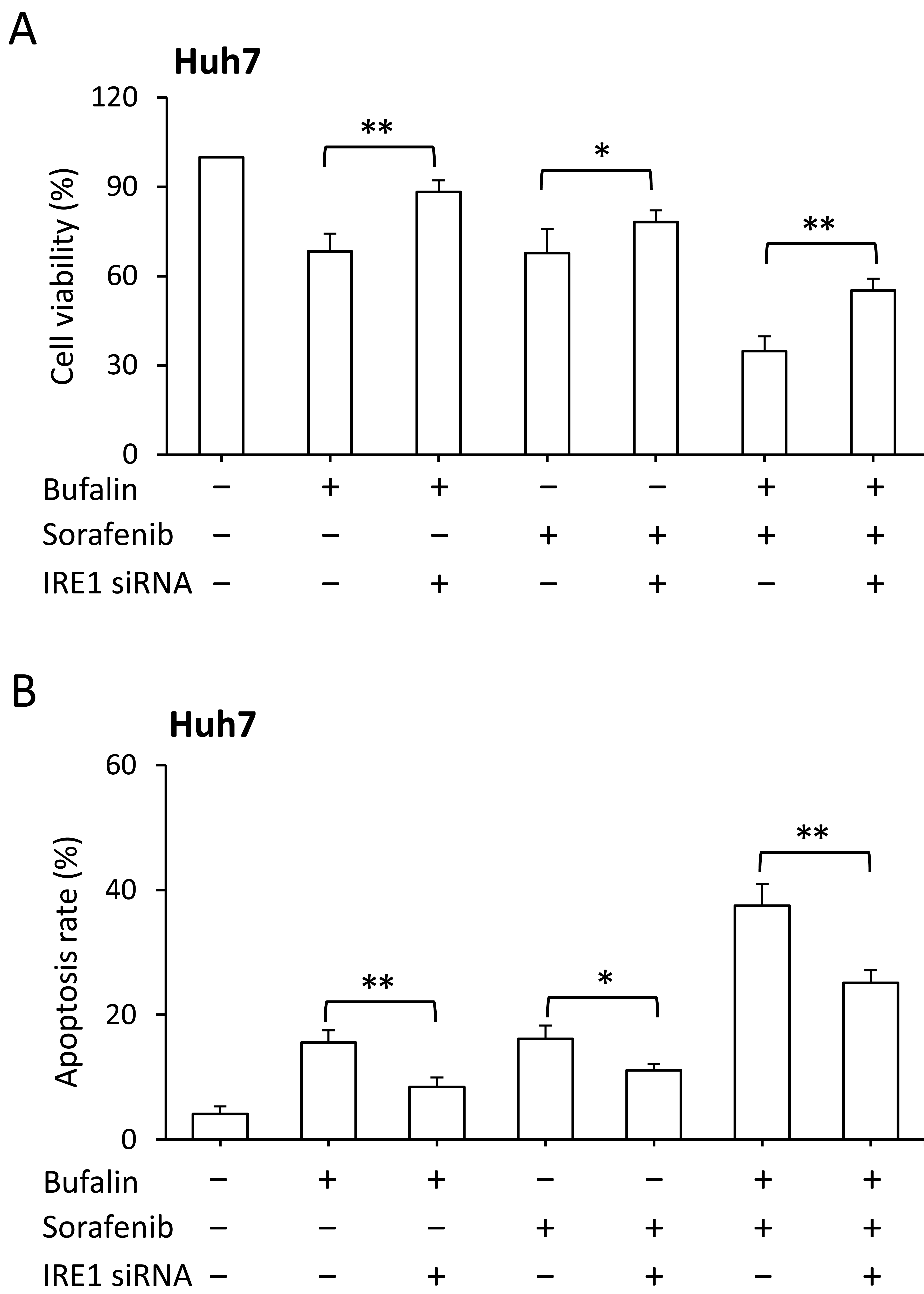

Supplement: S5 Fig — A-B, Huh7 cells were transfected with control or IRE1 siRNA for 24 h and then incubated with 100 nM bufalin, 5 μM sorafenib, or a combination of the two drugs for 48 h. (A) Cell viability (%) was measured. (B) The percentages of apoptotic cells (%) were measured by flow cytometry. Control siRNA-transfected cells served as controls. The relative band density from control cells was defined as 1. The data represent three independent experiments. “*” represents P<0.05, “**” represents P<0.001. (TIF) [file pone.0138485.s005.tif]

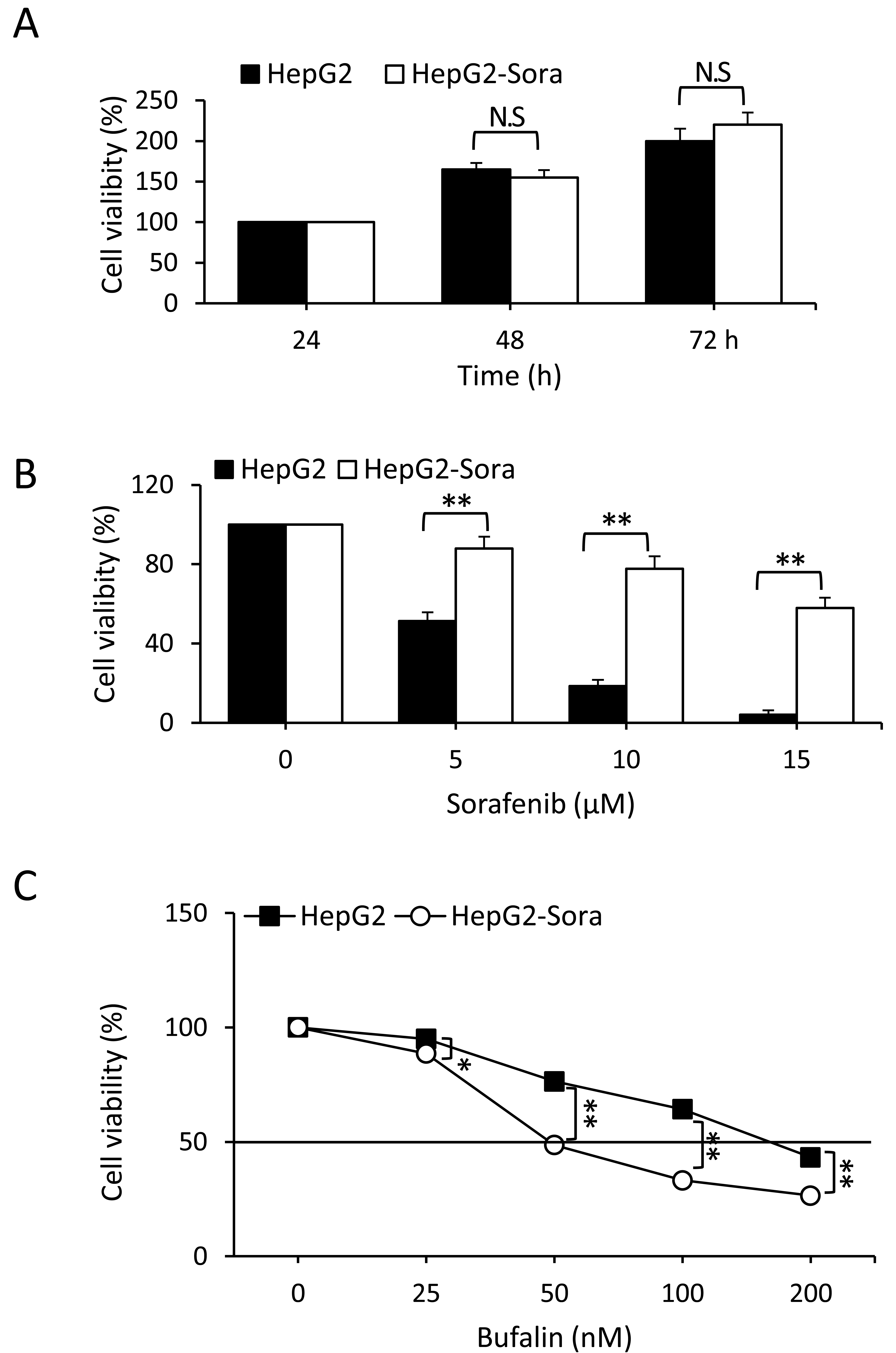

Supplement: S6 Fig — (A) HepG2 and HepG2-Sora cells were cultured in complete medium, and the viability was examined after 24, 48, and 72 h in culture. (B) The above cells were exposed to increasing concentrations of sorafenib for 48 h. Untreated cells served as controls. Cell viability (%) was compared with the corresponding untreated cells. (C) The above cells were exposed to increasing concentrations of bufalin for 48 h. Untreated cells served as controls. Cell viability (%) was compared with to the corresponding untreated cells. The data represent three independent experiments. N.S., not significant. The black line indicates the IC50. “*” represents P<0.05, “**” represents P<0.001. (TIF) [file pone.0138485.s006.tif]

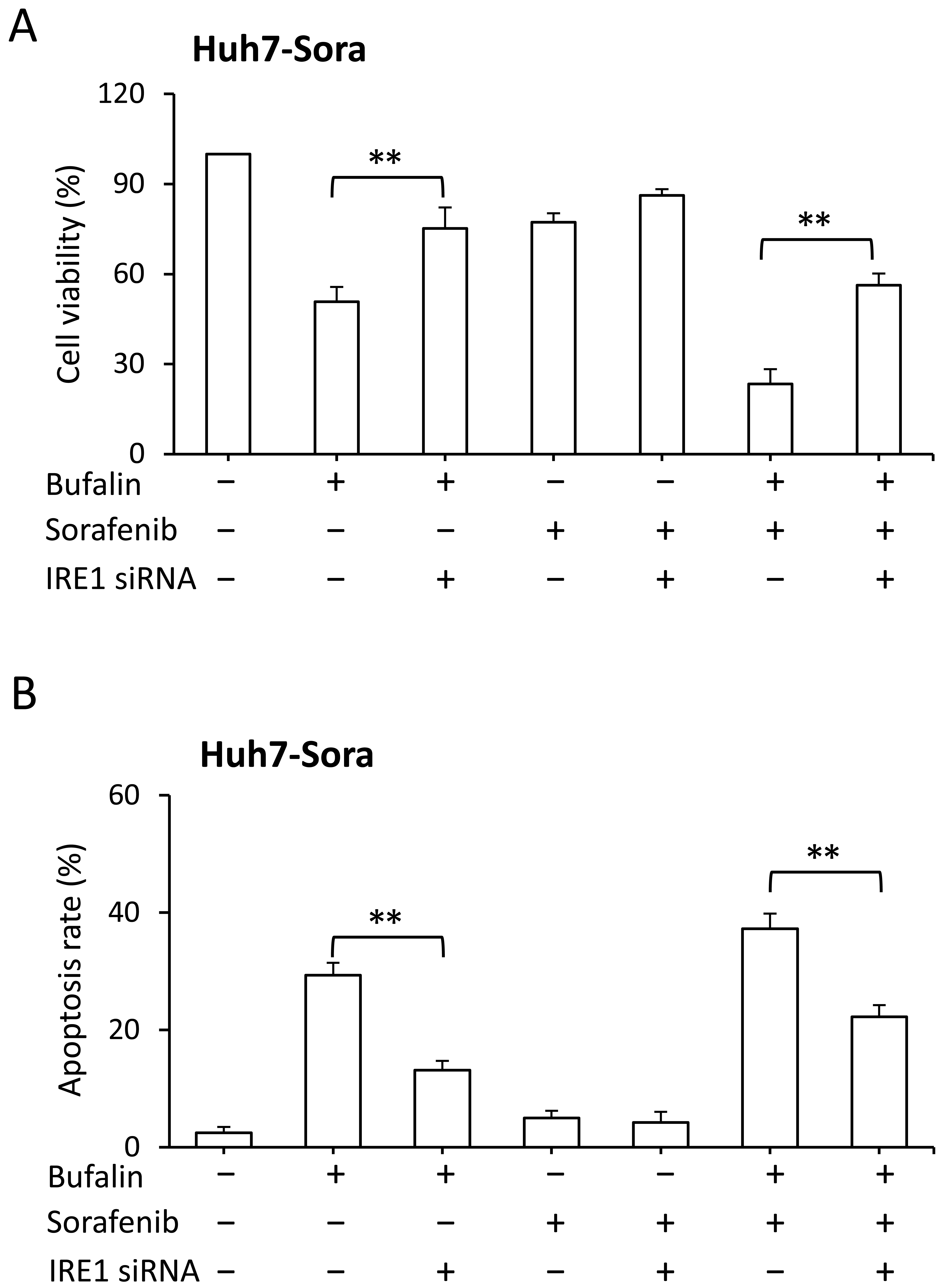

Supplement: S7 Fig — A-B, Huh7-Sora cells were transfected with control or IRE1 siRNA for 24 h and then incubated with 50 nM bufalin, 10 μM sorafenib, or a combination of the two drugs for 48 h. (A) Cell viability (%) was measured. (B) The percentages of apoptotic cells (%) were measured by flow cytometry. Control siRNA-transfected cells served as controls. The data represent three independent experiments. “**” represents P<0.001. (TIF) [file pone.0138485.s007.tif]
