# Supplementary material for: Bufalin Reverses Resistance to Sorafenib by Inhibiting Akt Activation in Hepatocellular Carcinoma: The Role of Endoplasmic Reticulum Stress
Source: PLoS One. 2015 Sep 18;10(9):e0138485. doi: 10.1371/journal.pone.0138485 (PMC4575108; doi:10.1371/journal.pone.0138485)
Supplement: S1 Table — (DOCX) [file pone.0138485.s008.docx]

**S1 Table. The CDIs of bufalin in combination with sorafenib in HepG2 cells.**

| Sorafenib (μM) | Bufalin (nM) | | | |
| --- | --- | --- | --- | --- |
|  | 25 | 50 | 100 | 200 |
| 2.5 | 0.92 | 0.93 | 0.87 | 0.96 |
| 5 | 0.85 | 0.84 | 0.70 | 0.85 |
| 10 | 0.95 | 0.94 | 0.87 | 0.93 |

Abbreviations: CDI, coefficient of drug interaction.
