# Supplementary material for: Bufalin Reverses Resistance to Sorafenib by Inhibiting Akt Activation in Hepatocellular Carcinoma: The Role of Endoplasmic Reticulum Stress
Source: PLoS One. 2015 Sep 18;10(9):e0138485. doi: 10.1371/journal.pone.0138485 (PMC4575108; doi:10.1371/journal.pone.0138485)
Supplement: S2 Table — (DOCX) [file pone.0138485.s009.docx]

**S2 Table. The CDIs of bufalin in combination with sorafenib in Huh7 cells.**

| Sorafenib (μM) | Bufalin (nM) | | | |
| --- | --- | --- | --- | --- |
|  | 25 | 50 | 100 | 200 |
| 2.5 | 0.94 | 0.91 | 0.88 | 0.95 |
| 5 | 0.96 | 0.83 | 0.74 | 0.94 |
| 10 | 0.95 | 0.95 | 0.77 | 0.96 |

Abbreviations: CDI, coefficient of drug interaction.
